# Supplementary material for: cGAMP Promotes Germinal Center Formation and Production of IgA in Nasal-Associated Lymphoid Tissue
Source: Med Sci (Basel). 2017 Dec 18;5(4):35. doi: 10.3390/medsci5040035 (PMC5753664; doi:10.3390/medsci5040035)
Supplement: Supplementary file 1 [file medsci-05-00035-s001.zip › medsci-247045-figures (2).pdf]

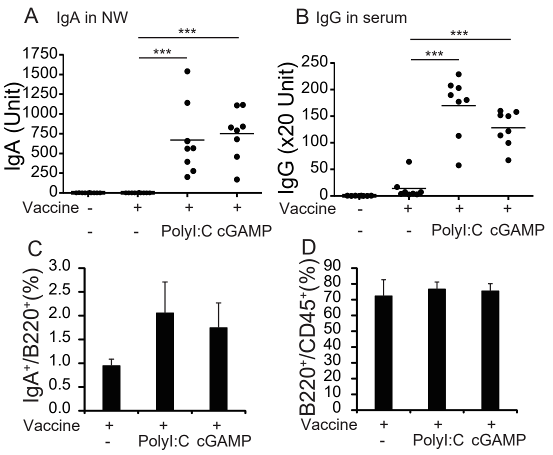

Takaki et. al., Figure 1

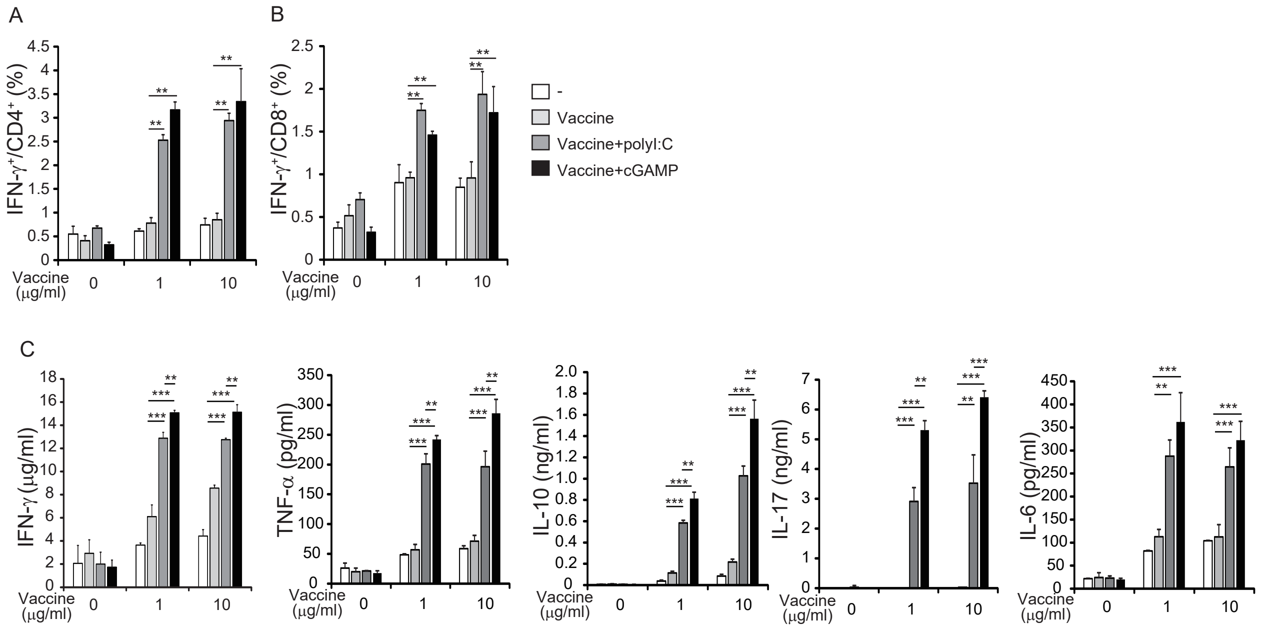

Takaki et. al., Figure 2

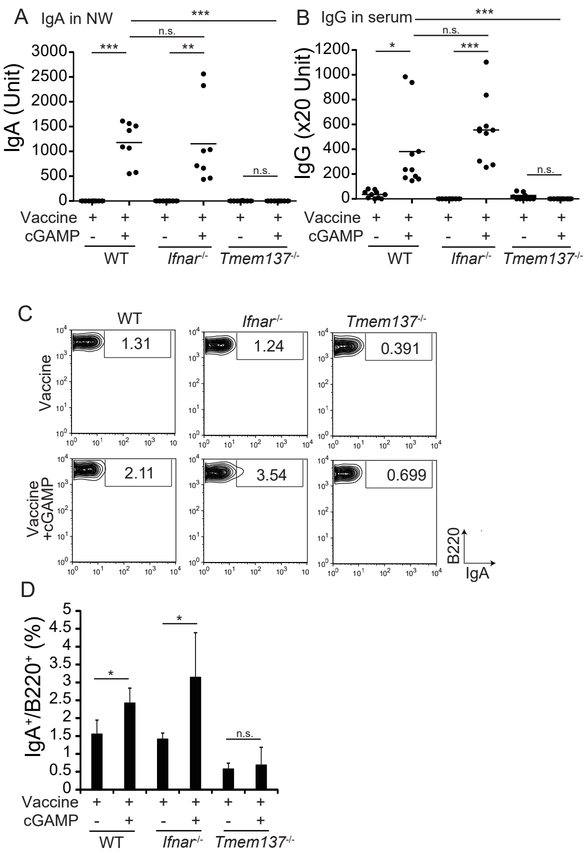

Takaki et. al., Figure 3

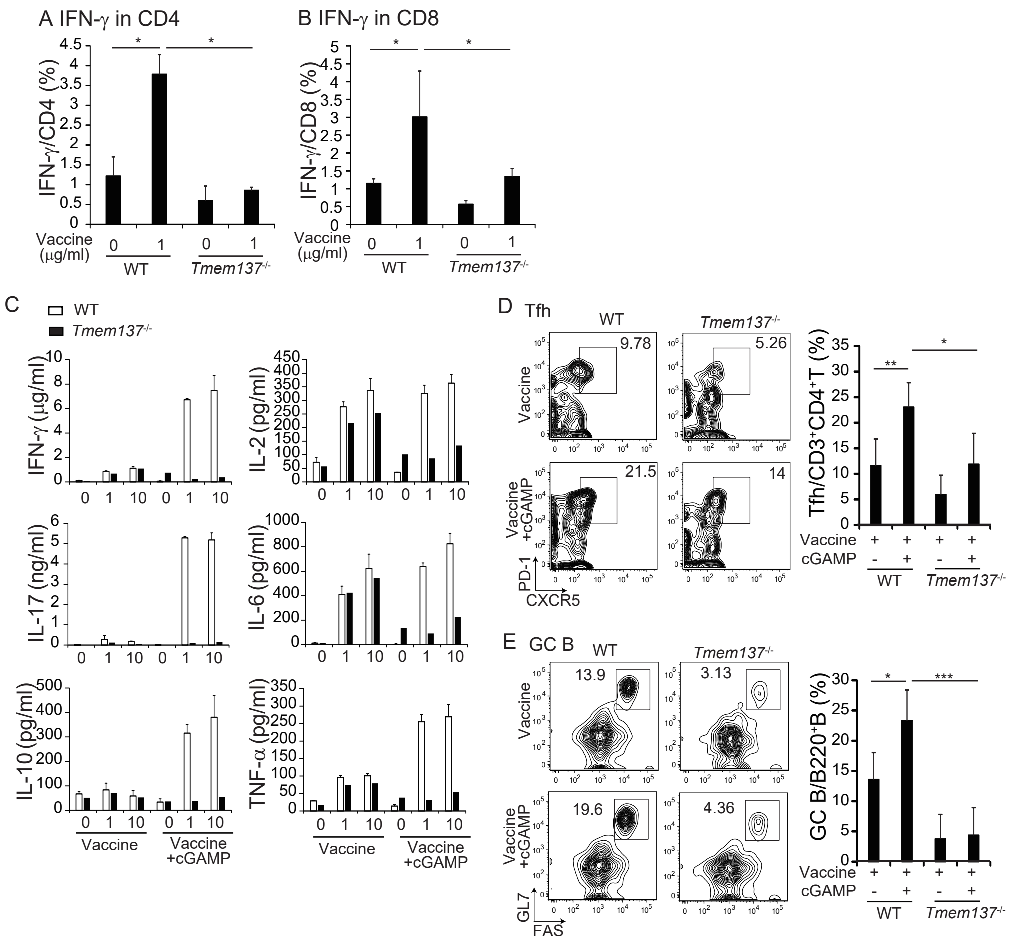

Takaki et. al., Figure 4

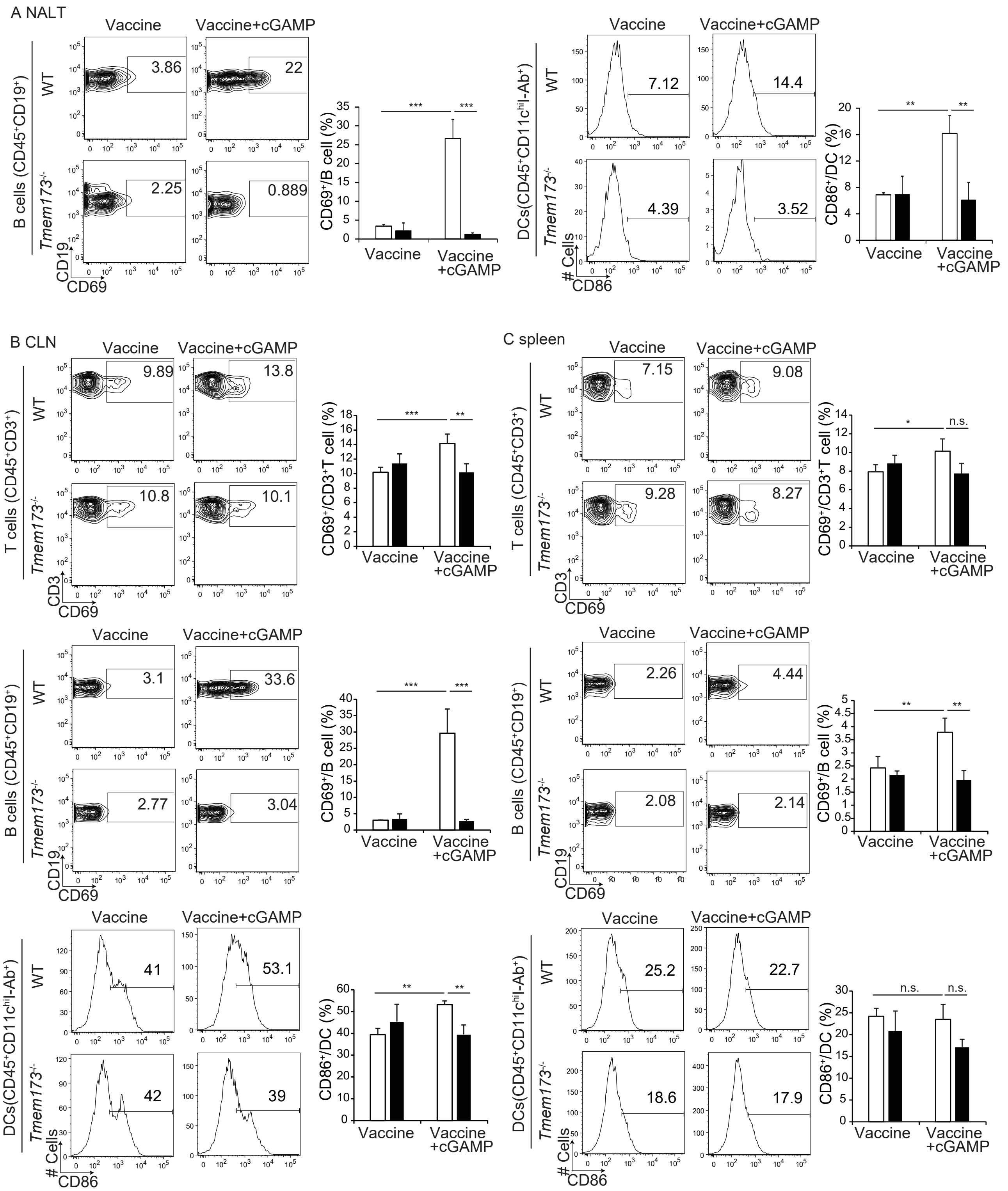

Takaki et. al., Figure 5

A

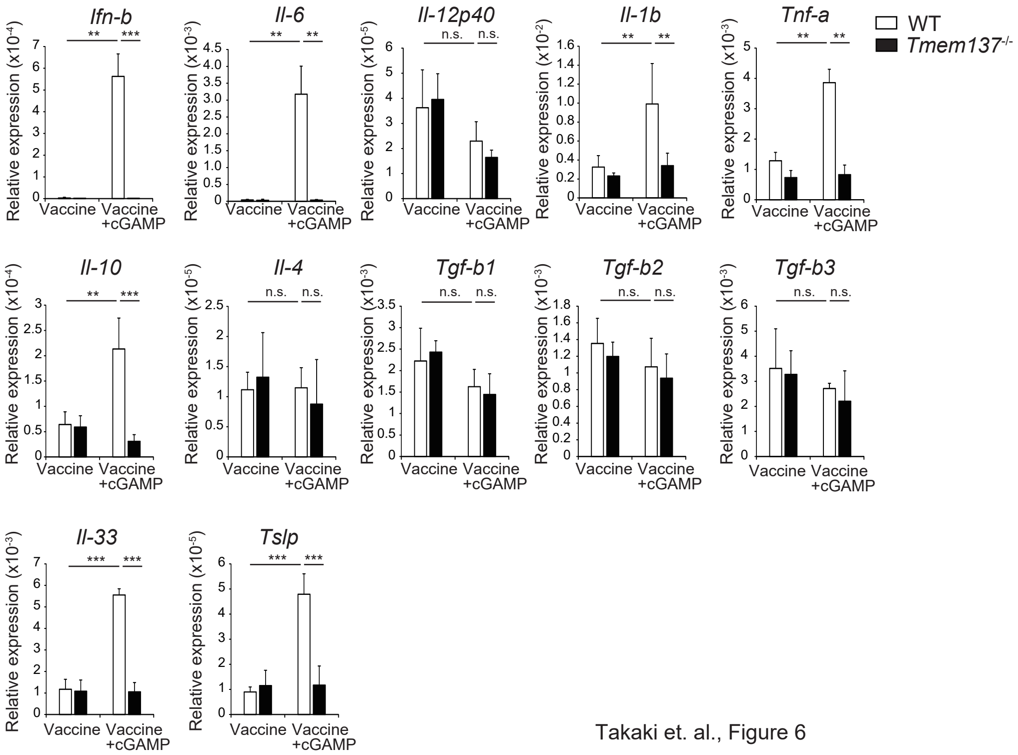

Takaki et. al., Figure 6

Supplementary Table S1. Primers' sequences

Primers' name	sequences (5'-3')
Il4 Fw	ggtctcaaccccccagctagt
Il4 Rv	gccgatgatctctctcaagtg
Il6 Fw	gttctctgggaaatcgtgga
Il6 Rv	tccagtttggtagcatccatc
Il10 Fw	ggcgctgtcatcgatttctc
Il10 Rv	tgctccactgccttgctctta
Ifnb Fw	ccagctccaagaaaggacga
Ifnb Rv	cgccctgtaggtgaggttgat
bactin Fw	tttgcagctccttcgttgc
bactin Rv	tcgtcatccatggcgaact
Tgf-b1 Fw	gctgaaccaaggagacggaat
Tgf-b1 Rv	caagagcagtgagcgctgaa
Tgf-b2 Fw	tcccgaataaaagcgaagagc
Tgf-b2 Rv	ggtgccatcaatacctgcaaa
Tgf-b3 Fw	ccagatacttcgaccggatga
Tgf-b3 Rv	tgacatcgaaagacagccattc
IL-12p40 Fw	aatgtctgcgtgcaagctca
IL-12p40 Rv	atgcccacttgctgcatga
Il1b Fw	tgacggaccccaaaagatga
IL1b Rv	tgctgctgcgagatttgaag
Tnfa Fw	aatggcctccctctcatcagt
Tfna Rv	gctacaggcttgtcactcgaatt
Il33 Fw	tcctgtctgtattgagaaacctga
Il33 Rv	tattttgcaaggcgggacca
Tslp Fw	ggactgtgagagcaagccag
Tslp Rv	tccgggcaaatgttttgtcg
